# Mg–O–F Nanocomposite Catalysts Defend against Global Warming via the Efficient, Dynamic, and Rapid Capture of CO_2_ at Different Temperatures under Ambient Pressure

**DOI:** 10.1021/acsomega.2c04587

**Published:** 2022-10-19

**Authors:** Samih A. Halawy, Ahmed I. Osman, Mahmoud Nasr, David W. Rooney

**Affiliations:** † Nanocomposite Catalysts Laboratory, Chemistry Department, Faculty of Science at Qena, South Valley University, Qena83523, Egypt; ‡ School of Chemistry and Chemical Engineering, Queen’s University Belfast, David Keir Building, BelfastBT9 5AG, Northern Ireland, U.K.

## Abstract

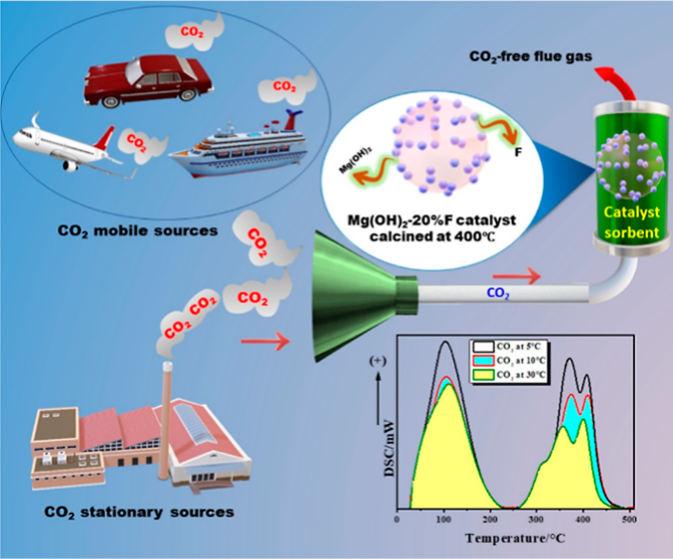

The utilization of Mg–O–F prepared from Mg(OH)_2_ mixed with different wt % of F in the form of (NH_4_F·HF), calcined at 400 and 500 °C, for efficient capture of CO_2_ is studied herein in a dynamic mode. Two different temperatures were applied using a slow rate of 20 mL·min^–1^ (100%) of CO_2_ passing through each sample for only 1 h. Using the thermogravimetry (TG)-temperature-programed desorption (TPD) technique, the captured amounts of CO_2_ at 5 °C were determined to be in the range of (39.6–103.9) and (28.9–82.1) mg_CO_2__·g^–1^ for samples of Mg(OH)_2_ mixed with 20–50% F and calcined at 400 and 500 °C, respectively, whereas, at 30 °C, the capacity of CO_2_ captured is slightly decreased to be in the range of (32.2–89.4) and (20.9–55.5) mg_CO_2__·g^–1^, respectively. The thermal decomposition of all prepared mixtures herein was examined by TG analysis. The obtained samples calcined at 400 and 500 °C were characterized by X-ray diffraction and surface area and porosity measurements. The total number of surface basic sites and their distribution over all samples was demonstrated using TG- and differential scanning calorimetry-TPD techniques using pyrrole as a probe molecule. Values of (Δ*H*) enthalpy changes corresponding to the desorption steps of CO_2_ were calculated for the most active adsorbent in this study, that is, Mg(OH)_2_ + 20% F, at 400 and 500 °C. This study’s findings will inspire the simple preparation and economical design of nanocomposite CO_2_ sorbents for climate change mitigation under ambient conditions.

## 1 Introduction

The world is on the verge of an environmental catastrophe due to global warming as a result of the continuous increase in atmospheric CO_2_. According to the data released by the Scripps Institute of Oceanography at the University of California in May 2022, the daily average concentration of atmospheric CO_2_ reached a record high of 421.37 parts per million (ppm).^1^ Numerous climate scientists and researchers are interested in experimenting with various CO_2_-capturing materials. Currently, the CO_2_ adsorption technique is used on some highly active materials, such as basic metal oxides, to combat global warming. In the pure form or promoted with other materials, MgO is regarded as a cornerstone component in all prepared materials for CO_2_ capture purposes, among these basic metal oxides.^2−9^ This characteristic relates to the abundance and strength of basic sites over the MgO surface.^10−12^ Other types of adsorbents, including the reduced graphene oxide–MnO_2_ nanocomposite,^13^ nickel–lithium silicate for CO_2_ capture and subsequent production of CH_4_,^14^ Li_4_SiO_4_-based sorbents for high-temperature CO_2_ capture,^15^ NiO-functionalized ultra-stable Y zeolite,^16^ Mg–Al mixed metal oxides for high-temperature CO_2_ capture,^17^ alumina-supported layered double hydroxides,^18^ CuAl_2_O_4_ nanoplates,^19^ Ni–CaO dual function materials,^20^ and CaO–Fe_2_O_3_–SiO_2_ composite, have been extensively examined.^21^ Few articles have focused on the study of capturing CO_2_ in a dynamic mode at different temperatures by flowing CO_2_ gas at different rates through the adsorbents.^22,23^ Many review articles with a forward-looking perspective discussed various CO_2_ capture techniques^24−27^ utilizing different adsorbent materials.

Rarely, published articles on using MgO–MgF_2_ mixtures for CO_2_ capture are cited in the literature. In this regard, this study can be viewed as proactive research into using these mixtures. Furthermore, pure MgF_2_ is considered to be a suitable and effective sintering additive for the preparation of certain types of ceramics,^28,29^ in manufacturing transparent IR windows,^30^ in solar thermal technology,^31^ and as a catalyst in the form of MgF_2–*x*_(OH)_*x*_ during aldol condensation of furfural and acetone.^32^

On the occasion of the 27th UN Climate Change Conference of the Parties (COP27), which will be held in Sharm el-Sheikh, Egypt, in November 2022, two research teams from South Valley University (Egypt) and Queen’s University Belfast (UK) collaborated in a vital effort to combat global warming. Herein, our samples were prepared using a direct and simple method and exhibited a relatively high surface area and a superior reactivity toward CO_2_ capture at 5 and 30 °C, despite a limited CO_2_ flow time (1 h) throughout the samples. The applied method is based on two environmentally friendly materials, that is, Mg(OH)_2_ and NH_4_F·HF, which were used to prepare the required mixtures without releasing harmful greenhouse gases.

Early on, it was determined that the generation of MgCO_3_ from MgO without a promoter has poor kinetics and is limited to a few surface carbonate layers, resulting in only a small fraction (<2%) of the theoretical CO_2_ sorption capacity of MgO. The slowdown kinetics is caused by the high lattice enthalpy of MgO and the formation of a layer of magnesium carbonate, which acts as a barrier for CO_2_ molecules. Alkali metal salts are the most effective promoters for MgO-based sorbents.^2^ This work focuses on the preparation of Mg–O–F nanocomposite catalysts with a high surface area, a large population of surface basic sites, and a high CO_2_ capture efficiency using environmentally friendly materials without emitting harmful greenhouse gases. Compared to recently published articles utilizing other adsorbents, these samples appear to be more effective CO_2_ adsorbents under ambient conditions.

## 2 Experimental

### 2.1 Preparation of Mg–O–F Nanocomposite Mixtures

Mixed samples of Mg(OH)_2_ with 20–50% F (by weight) as ammonium hydrogen difluoride (AHDF) NH_4_F·HF were prepared as follows: calculated amounts of ammonium hydrogen difluoride (NH_4_F·HF, Hopkin & Williams) were dissolved in deionized water in Teflon beakers and mixed well with the corresponding weights of Mg(OH)_2_ (BDH, chemicals). These mixtures were then evaporated until dry in a water bath. The resulting mixtures were dried at 100 °C overnight. All these mixtures were also calcined at two temperatures of 400 and 500 °C in static air for 3 h.

### 2.2 Characterization

A 50H thermogravimetric analyzer and differential scanning calorimetry (DSC) instruments (Shimadzu—Japan) were used in this work. A slow heating rate of 3 °C min^–1^ was used in the He atmosphere (40 mL·min^–1^) during the study of the thermal stability of all prepared mixtures, as well as the pure compounds of Mg(OH)_2_ and NH_4_F·HF. Thermogravimetry (TG) and DSC experiments of the temperature-programed desorption (TPD) of CO_2_ over all samples under study, at different temperatures of CO_2_ adsorption, were accomplished using a heating rate of 10 °C min^–1^ in a dynamic N_2_ flow (40 mL·min^–1^). The instrument is equipped with a data acquisition and handling system (TA-50WSI), and highly sintered α-Al_2_O_3_ was applied as reference material in DSC experiments.

X-ray diffraction (XRD) patterns of the calcined samples at 400 and 500 °C were measured by powder XRD using a Brucker AXS-D8 Advance diffractometer (Germany), equipped with a copper anode generating Ni-filtered CuKa radiation (*k* = 1.5406 Å) from a generator operating at 40 kV and 40 mA in the 2θ range between 10 and 80°. The instrument is supported with interfaces of *DIFFRAC*^*plus*^ SEARCH and *DIFFRAC*^*plus*^ EVA to facilitate an automatic search and match of the crystalline phases for identification purposes with the ICDD database.

The Brunauer–Emmett–Teller (BET) surface area and porosity of all samples calcined at 400 and 500 °C were measured by N_2_ adsorption/desorption isotherms at the liquid nitrogen temperature (−196 °C) using a Micromeritics ASAP 2020 system, equipped with an online data acquisition and handling system operating BET and Barrett–Joyner–Halenda analytical software. All samples were degassed at 200 °C and 10^–5^ Torr for 2 h before measurements (1 Torr = 133.3 Pa).

The total number of basic sites (sites·g^–1^) over each sample was measured by the TPD of pyrrole (99%-ACROS Organics, New Jersey, USA) as a probe molecule using TG and DSC techniques. The experimental details can be explained as follows:^33,34^ 60 mg of each sample is preheated at 390 °C for 1 h in the air before the probe molecule is exposed. 20 ± 2 mg covered samples with pyrrole were subjected to thermogravimetric analysis (TGA) and another sample was subjected to DSC analyses at a heating rate of (10 °C·min^–1^) in a dry N_2_ flow (40 mL·min^–1^), using a 50H Shimadzu thermal analyzer (Japan). The thermal analyzer is equipped with a data acquisition and handling system (TA-50WSI). α-Al_2_O_3_ was used as the reference material in DSC measurements. The mass loss due to the desorption of pyrrole during TG experiments from the basic sites was determined to measure the total surface basicity as sites per gram. Equation 1 is used to estimate the total number of surface basic sites (*N*_basic_):^35^
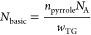
1where *n*_pyrrole_ is the number of moles of desorbed pyrrole, *N*_A_ is Avogadro’s number (sites/mol), and *w*_TG_ is the weight of the TG sample (g).

### 2.3 CO_2_ Capture Experiments in a Dynamic Mode under Ambient Pressure

**Figure 1:**
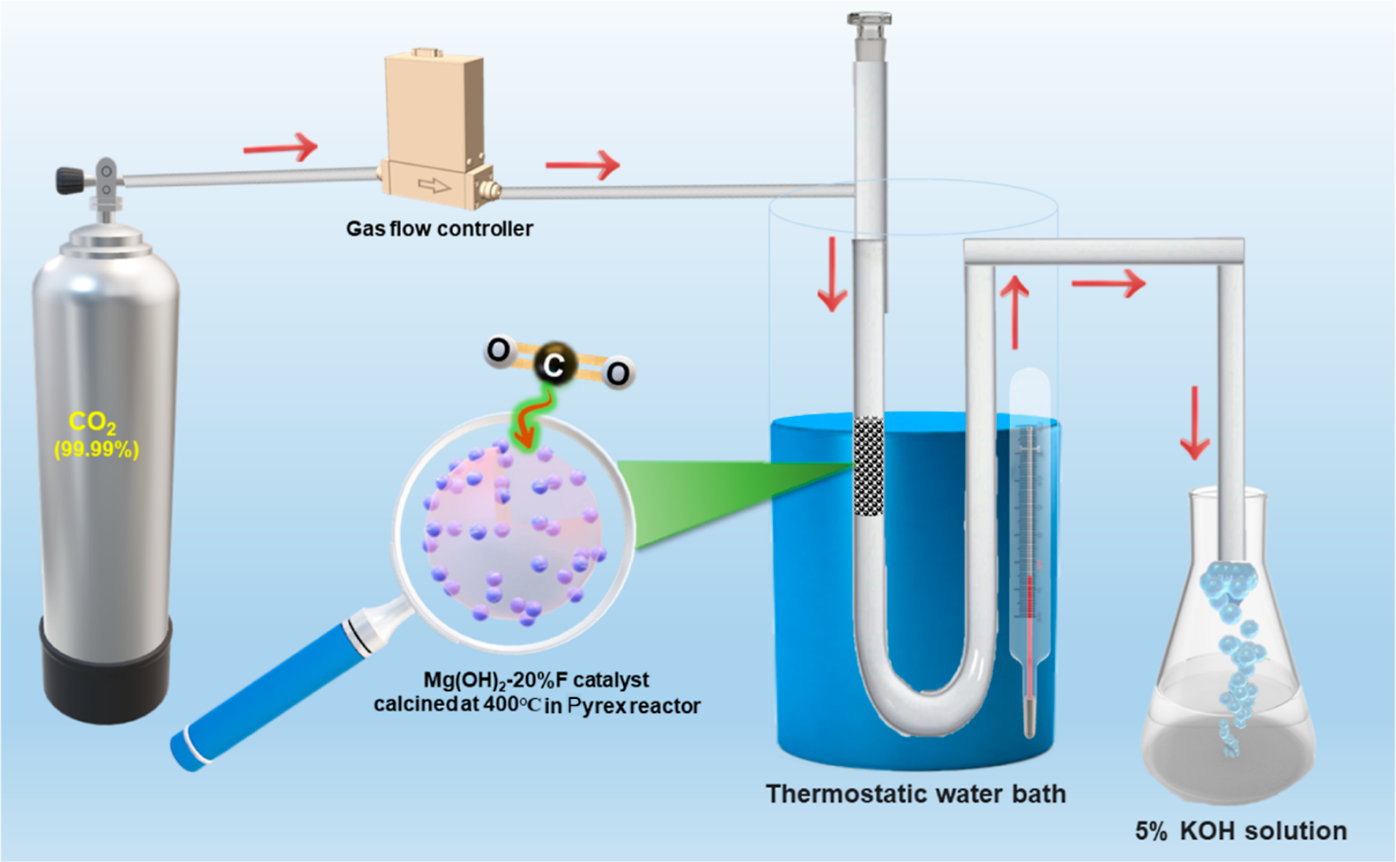
Schematic diagram of CO_2_ captured in a dynamic mode at different temperatures with a flow rate of 20 mL·min^–1^.

Before the CO_2_ capture experiment, each sample was activated in a homemade fixed-bed U-tube Pyrex reactor by heating 100 mg of the sample at 390–400 °C for 2 h in an oxygen flow (150 mL·min^–1^). The sample is cooled down to room temperature (RT), and the reactor is then transferred to a thermostatic water bath set to the desired temperature, that is, at 5, 10, or 30 °C, as shown in Figure 1. The CO_2_ gas stream (99.9%) is allowed to pass over the catalyst bed for 1 h, with a flow rate = 20 mL·min^–1^, permeating between catalyst particles inside the reactor to exit from the opposite side of the reactor. The residual CO_2_ flow is trapped by passing through an aqueous 5% solution of KOH. The quantity of CO_2_ molecules captured by each sample was measured immediately, employing the TPD technique, using the above-mentioned TG and DSC units as follows: 20–25 mg of each sample was subjected to TG and DSC analyses on heating to 425 °C, in the case of samples calcined at 400 °C, or to 525 °C in the case of the other samples treated at 500 °C (at a 10 °C·min^–1^ heating rate) in dry N_2_ (flow rate = 40 mL·min^–1^). In TG curves, the mass loss due to CO_2_ desorption was measured as the capturing efficiency of each sample at the specified temperature. Values of the molar enthalpy change (Δ*H*_des_ in J mol^–1^) corresponding to the desorption of CO_2_ in each step, at different temperatures, of the most active samples were calculated from the DSC curves.^36^

## 3 Results and Discussion

### 3.1 Thermogravimetric Analysis

**Figure 2:**
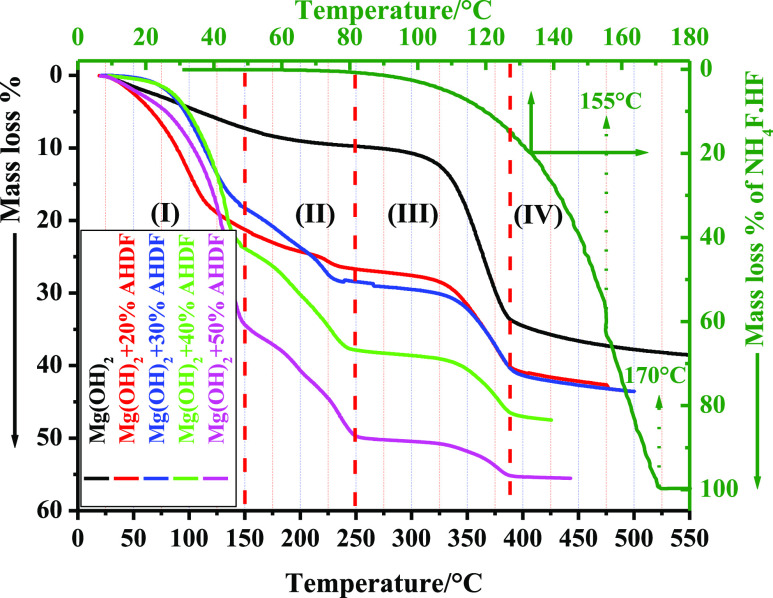
TG curves of Mg(OH)_2_, NH_4_F·HF (AHDF), and their mixtures were performed in 40 mL·min^–1^ He, with a 3 °C·min^–1^ heating rate.

A slow heating rate was chosen (3 °C·min^–1^) to examine the thermal decomposition of both Mg(OH)_2_ and ammonium hydrogen difluoride (AHDF) NH_4_F·HF as pure precursors, as well as all their mixtures in the He atmosphere, as presented in Figure 2. Due to the loss of physically adsorbed water molecules, Mg(OH)_2_ began to lose weight upon heating immediately and continuously up to 200 °C. This step is followed by another weight loss at approximately 270 °C, which is ascribed to the dehydroxylation of Mg(OH)_2_.^37,38^ At 400 °C, the calculated mass loss was 34.5%, whereas at 500 °C, it was 37.7%. Due to the liberation of physically adsorbed water molecules associated with Mg(OH)_2_, these values exceeded the theoretical value^38−40^ for the conversion of anhydrous Mg(OH)_2_ to MgO (ca. 30.86%), as presented in eq 2:
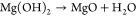
2on the other hand, NH_4_F·HF undergoes melting at 125 °C, followed by a subsequent fast step due to its decomposition around 150 °C,^41^ as shown in eq 3:
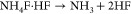
3the TG profiles of the mixed samples can be divided into four temperature zones, as shown in Figure 2. The first zone in the temperature range RT–150 °C, includes both melting and decomposition of AHDF (see eq 3). Zone-II in the temperature range 150–250 °C exhibits the reaction of the HF liberated by eq 3 with Mg(OH)_2_ in these mixtures to produce magnesium fluoride and magnesium fluoride hydroxide^42^ as follows:
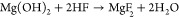
4and
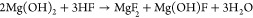
5Notably, the % mass loss associated with this step in Zone-II increased from 5.1 to up to 15.1% as the % (*x*) of AHDF added to Mg(OH)_2_ increased from 20 to 50%, respectively. This may be attributable to the increased amounts of the liberated HF that participate in reactions 4 and 5 in these mixtures. Furthermore, melting NH_4_F·HF as a fluorinating agent in these mixtures facilitates the formation of both MgF_2_ and magnesium fluoride hydroxide Mg(OH)F at a low-temperature range in all prepared samples.^42,43^ It is a benefit of this method that MgF_2_ can be prepared more easily. This phenomenon has a negative impact on these samples’ surface area, as will be discussed later. The third zone (250–390 °C) could be related to both dehydroxylation and dehydration processes,^42^ followed by a slow weight loss step at a temperature above 390 °C (Zone-IV) due to the removal of any residual H_2_O molecules still bonded on the surface of the solid samples, as follows:
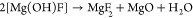
6according to the TG curves of the last two samples, the final product at 400 °C, Mg(OH)_2_-40% F and Mg(OH)_2_-50% F, suggests the formation of the Mg–O–F nanocomposite as a mixture of MgF_2_ and MgO. XRD analysis will provide further explanation to confirm this hypothesis.

### 3.2 XRD Analysis

**Figure 3:**
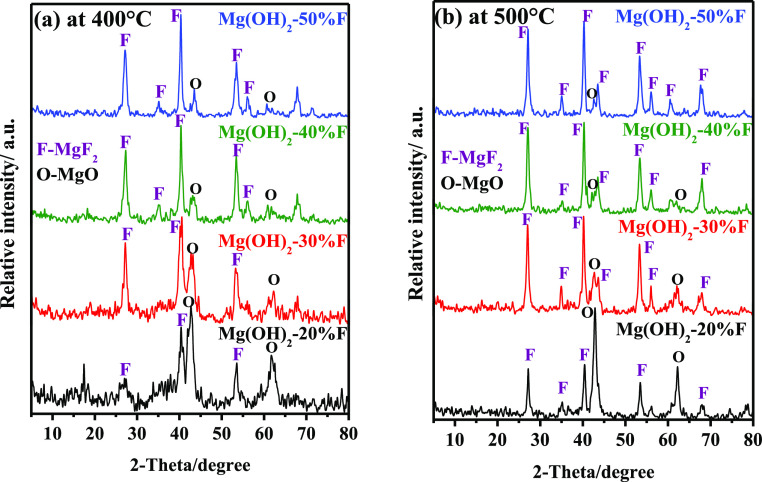
XRD patterns of Mg(OH)_2_ mixed with 20–50% F (wt by wt) calcined in static air for 3 h at (a) 400 and (b) 500 °C.

XRD analysis of the prepared nanocomposite mixtures at 400 and 500 °C was performed in the range of 10–80° (2θ) to confirm the nanocomposite structure of these samples. Figure 3a,b demonstrates the XRD patterns of all samples. The diffraction patterns of all samples calcined at 400 °C (Figure 3a) showed two main diffraction peaks belonging to the polycrystalline cubic structure of MgO (ICCD file: 74-1225)^12,44,45^ at 42.785 and 62.233°, with Miller indices values of (200) and (220), respectively. These diffraction peaks were sharp and predominant in the case of Mg(OH)_2_-20% F, then their intensity decreased gradually with increasing (*x*) % F added to Mg(OH)_2_, see Figure 3a. On the other hand, a group of diffraction peaks associated with the formation of tetragonal MgF_2_ (ICCD file: 6-0290) in these mixtures^43,46−49^ was recorded along with the crystallographic planes (110), (101), (111), (201), (211), and (220), which correspond to the Bragg reflections at 2θ values of 27.265, 35.174, 40.405, 43.613, 53.468, and 56.149°, respectively. The sharpness and intensity of these diffractions increased steadily from Mg(OH)_2_-20% F up to Mg(OH)_2_-50% F due to the formation of further MgF_2_ in these nanocomposite mixed samples. The same diffraction peaks were recorded in all patterns of samples calcined at 500 °C, as shown in Figure 3b, as previously explained in the samples calcined at 400 °C. These diffractions were characterized by a strong intensity and sharpness. Furthermore, the predominance of MgF_2_ was clearly observed in these samples at 500 °C, particularly in those containing more than 20% F, see Figure 3b. The XRD analysis of our samples reveals that the Mg(OH)_2_-20% F sample, whether calcined at 400 or 500 °C, contains more MgO than MgF_2_. This phenomenon will significantly impact the sample’s surface area and CO_2_ capturing efficiency, as will be discussed later. The mean crystallite size of MgO in all samples was calculated using the (200) reflection to be within the range of 7.9–9.4 nm and between 8.6 and 12.0 nm for samples calcined at 400 and 500 °C, respectively. Furthermore, the crystallite size of MgF_2_ in all samples was estimated using the main reflection (111) and was in the range of 3.2–19.1 and 21.6–26.7 nm for samples calcined at 400 and 500 °C, respectively. The calculated values of the crystallite size using the Scherrer equation of all samples are cited in Table 1. Notably, increasing the calcination temperature from 400 to 500 °C resulted in the formation of larger MgO and MgF_2_ crystallite phases in all samples.

**Table 1:** Textural Properties of Mg(OH)_2_ Modified with *x* % F (by wt) That Was Calcined at 400 and 500 °C for 3 h in Air and the Crystallite Size of the Obtained Phases, as Calculated from XRD Patterns

	XRD crystallite size (nm)^a^							
adsorbent composition	MgO* cubic	MF_2_^**^ tetragonal	*S*_BET_(m^2^/g)	external surface area (m^2^/g)	micropore area (m^2^/g)	total pore volume (cm^3^/g)	mesopore volume (cm^3^/g)	average pore diameter (nm)
Mg(OH)_2_ + 20% F-400	9.4	3.2	153.22	145.79	7.43	0.23	0.23	3.7
Mg(OH)_2_ + 30% F-400	7.9	8.9	117.18	111.49	5.68	0.26	0.26	3.7, 13.0
Mg(OH)_2_ + 40% F-400	8.6	14.6	88.27	79.39	8.88	0.29	0.29	3.6, 16.0
Mg(OH)_2_ + 50% F-400		19.1	72.22	61.59	10.62	0.31	0.30	3.6, 18.9
Mg(OH)_2_ + 20% F-500	12.0	22.7	103.06	96.25	6.81	0.24	0.24	3.5, 6.1
Mg(OH)_2_ + 30% F-500	8.6	22.8	81.93	80.74	1.18	0.16	0.16	3.6
Mg(OH)_2_ + 40% F-500	9.9	21.6	58.32	54.31	4.00	0.13	0.13	3.7, 33.0
Mg(OH)_2_ + 50% F-500		26.7	39.70	33.83	5.87	0.12	0.11	3.5, 33.8

a The position of the most intense peak is at (*) 2θ = 42.84° (200) in the case of MgO and (**) = 40.38° (111) in the case of MgF_2_.

### 3.3 Surface Area and Porosity Measurements

**Figure 4:**
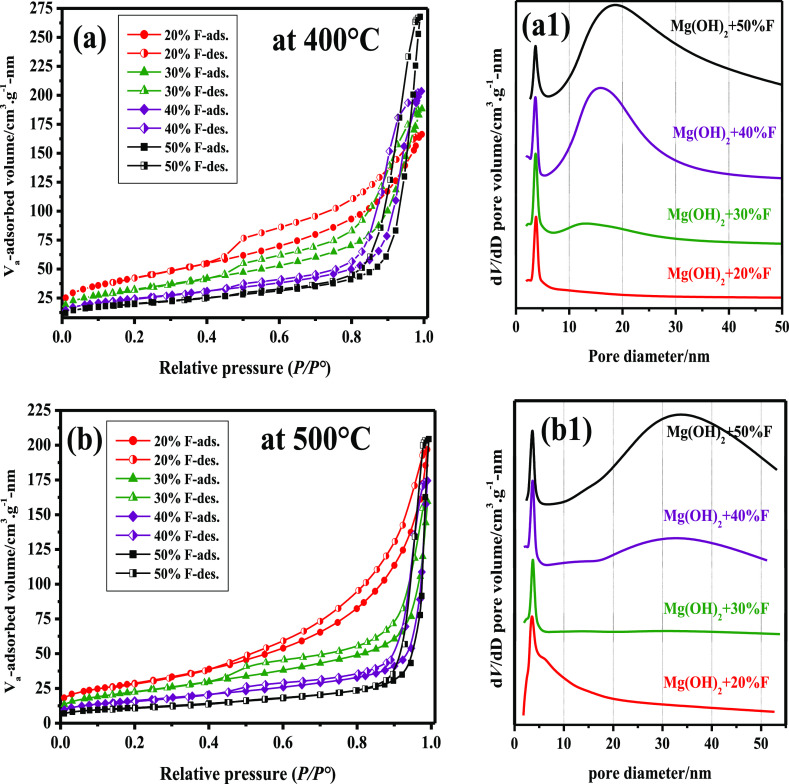
N_2_ adsorption–desorption isotherms and pore diameter profiles of samples of Mg(OH)_2_-(*x*) wt % F calcined at 400 °C [a,a(1)] and at 500 °C [b,b(1)].

The textural properties of the samples calcined at 400 and 500 °C were investigated using the BET method. Figure 4 exhibits the N_2_ adsorption/desorption isotherms that were recorded at −196 °C and the pore volume profiles of all samples. It is concluded from Figure 4a,b that all samples, whether calcined at 400 or 500 °C, display type IV isotherms, as recommended by the IUPAC,^50,51^ with type H3 hysteresis loops.^52^ The calculated values of the surface area (*S*_BET_) and the corresponding values of the external surface area of samples calcined at 400 °C, as shown in Table 1, were approximately 1.4–1.5 times higher than those calcined at 500 °C, except for samples containing 50% F, which experienced a clear reduction in the surface area. The gradual decrease of the surface area of all samples with the increase in the added amounts of (*x*)% F as NH_4_F·HF is probably due to the melting of this fluorinating agent^41^ at an early stage in the calcination process of these mixtures. This phenomenon has caused the surface area of all samples calcined for 3 h at 400 and 500 °C to decrease. The adsorption hysteresis of all samples was located in the *P*/*P*° region of 0.40–0.45, except for one sample, that is, Mg(OH)_2_ mixed with 50% F and calcined at 500 °C, see Figure 4b. This is interpreted as evidence that all samples are mesoporous.^51^ The pore volume profiles of these samples strongly supported these findings; see Figure 4a(1),b(1). All samples showed pores with a 3.5–3.7 nm diameter, see Table 1. Increasing the mixing ratios of (*F*), that is, > 20% F, has resulted in the generation of new wide pores with diameters of 13, 16, and 18.9 nm in the case of samples calcined at 400 °C. Two samples, namely, Mg(OH)_2_-40% F and Mg(OH)_2_-50% F, calcined at 500 °C, recorded wider mesopores with diameters equal to 33 and 33.8 nm, respectively, see Figure 4a(1),b(1). Finally, the calculated values of both the total pore volume and mesopore volume in cubic centimeter per gram, as shown in Table 1, varied steadily with the addition of (*x*) % F in these nanocomposite mixtures. This is due to the release of more NH_3_ during the decomposition of molten NH_4_F·HF, as shown in eq 3, resulting in larger pore volumes in samples calcined at 400 °C. In contrast, samples calcined at 500 °C exhibited a gradual decrease in the total pore volume and mesopore volume. This may be a result of the formation of larger crystallites at 500 °C at the expense of the pore volumes in these mixtures, see Table 1.

### 3.4 Assessment of the Surface Basicity Using TG and DSC-TPD Techniques of Pyrrole

The surface basicity of any adsorbent is critical for achieving a high catalytic activity in the dehydrogenation reaction of alcohols^53^ and enhancing its capacity to capture CO_2_.^54^ The catalytic surface with the highest concentrations of basic sites is the most effective in capturing CO_2_. Using TG- and DSC-TPD techniques, pyrrole was used as a probe molecule to determine the number and strength of surface basic sites over all samples in this work. Numerous articles have studied the TPD technique of pyrrole^55−57^ to categorize the different types of basic sites over solid catalysts.

Others investigated the use of pyrrole as an IR spectroscopic molecular probe in a surface basicity determination of MgF_2_ and metal oxides,^58,59^ as well as employing pyrrole as an NMR probe molecule to determine the basic sites’ strength over solid catalysts.^60^ Very recently, Chen and his co-workers^33^ proposed some possible intermediates of pyrrole due to its reactions with the O/Cu (100) surface, based on the TPD curves of pyrrole.

**Figure 5:**
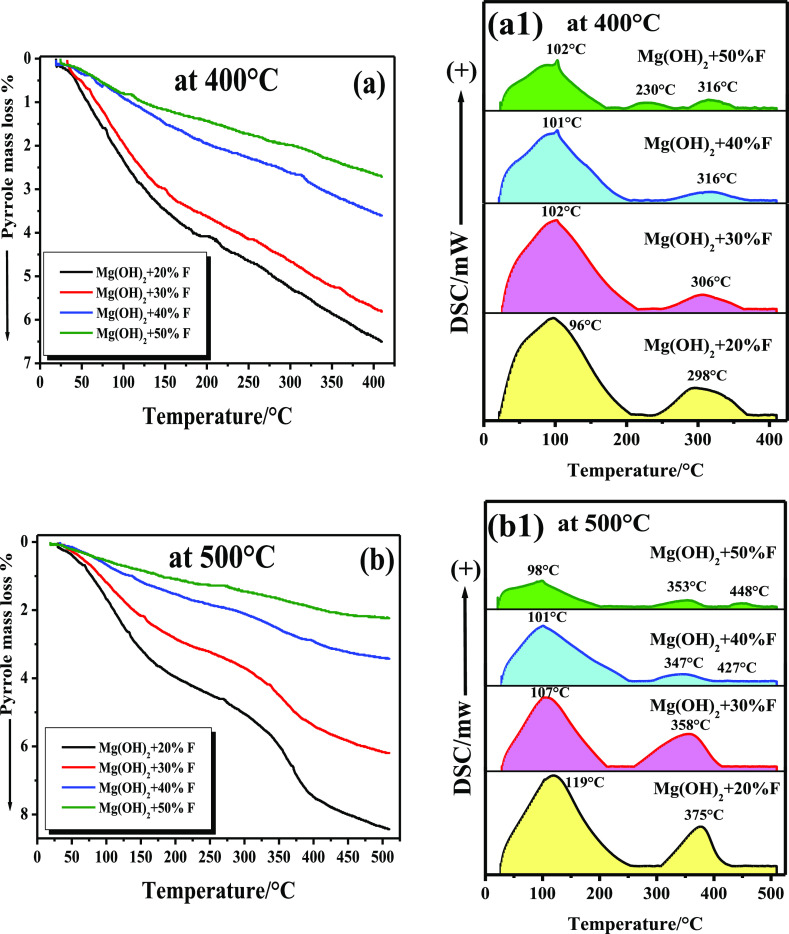
Pyrrole TG and DSC-TPD curves recorded for Mg(OH)_2_ mixed with 20–50% (wt/wt) F, as NH_4_·HF, calcined at 400 °C [a,a(1)] and calcined at 500 °C [b,b(1)].

TG and DSC-TPD profiles of pyrrole recorded for all samples, calcined at 400 and 500 °C, are shown in Figure 5. The total number of basic sites over each sample is calculated from the TG curve, while the DSC profile exhibits the distribution and strength of the different types of these basic sites over its surface. The weight loss due to the desorption of pyrrole from the surface of each sample, in milligrams or pyrrole per gram and the total number of molecules of pyrrole desorbed per gram corresponding to the indicated temperature range were calculated and are shown in Table 2. Increasing the (*x*) % F from 20 to 50% added to Mg(OH)_2_ in these mixtures was accompanied by the formation of more MgF_2_ in these samples, as shown in XRD patterns, see Figure 3. It is known that MgF_2_ particles possess a strong Lewis acidity during their formation in magnesium hydroxyl fluoride.^58,61^ Consequently, a steady reduction of the total basic sites can be observed through these samples, whether calcined at 400 or 500 °C, as shown in the TG curves (Figure 5a,b) and the calculated values in Table 2. The calculated values of all samples’ total number of basic sites are fully compatible with the surface area measurement, see Table 2. Observing the DSC-TPD profiles of samples calcined at 500 °C, shown in Figure 5b(1), reveals that the basic sites over these samples are more pronounced than those over samples calcined at 400 °C. The recorded *T*_max_ values of the strong basic sites, over samples calcined at 500 °C, were shifted to higher temperatures in the range of 347–375 °C. Furthermore, a new type of strong basic sites clearly appeared in the case of Mg(OH)_2_-50% F that was calcined at 400 and 500 °C at higher temperatures, see Figure 5a(1),b(1). From the results mentioned above, it is notable that all samples contain a higher ratio of weak basic sites, in addition to strong basic sites with a low ratio at higher temperatures, see the DSC-TPD profiles in Figure 5a(1),b(1). As is discussed in the following section, this distribution of basic sites will further support samples’ efficiency in capturing CO_2_ at low temperatures.

**Table 2:** Basicity Measurements Calculated Based on TG-TPD Curves of Pyrrole over Mg(OH)_2_ + *x* % F Adsorbents Calcined, for 3 h in Air, at 400 and 500 °C

	calcined at 400 °C			calcined at 500 °C		
adsorbent	*S*_BET_(m^2^·g^–1^)	apyrrole RT–400 °C(mg_pyrr_·g^–1^)	btotal no. of basic sites(site·g^–1^)	*S*_BET_(m^2^·g^–1^)	apyrrole RT–500 °C(mg_pyrr_·g^–1^)	btotal no. of basic sites (site·g^–1^)
Mg(OH)_2_ + 20% F	153.22	62.5	5.61 × 10^20^	103.06	82.4	7.39 × 10^20^
Mg(OH)_2_ + 30% F	117.16	56.7	5.09 × 10^20^	81.93	60.2	5.40 × 10^20^
Mg(OH)_2_ + 40% F	88.27	35.1	3.15 × 10^20^	58.32	33.4	3.00 × 10^20^
Mg(OH)_2_ + 50% F	72.22	26.3	2.36 × 10^20^	39.7	21.6	1.93 × 10^20^

a Mass loss calculated from TG-TPD curves.

b Total number of basic sites (sites per g_solid_).

### 3.5 CO_2_ Capture Study

**Figure 6:**
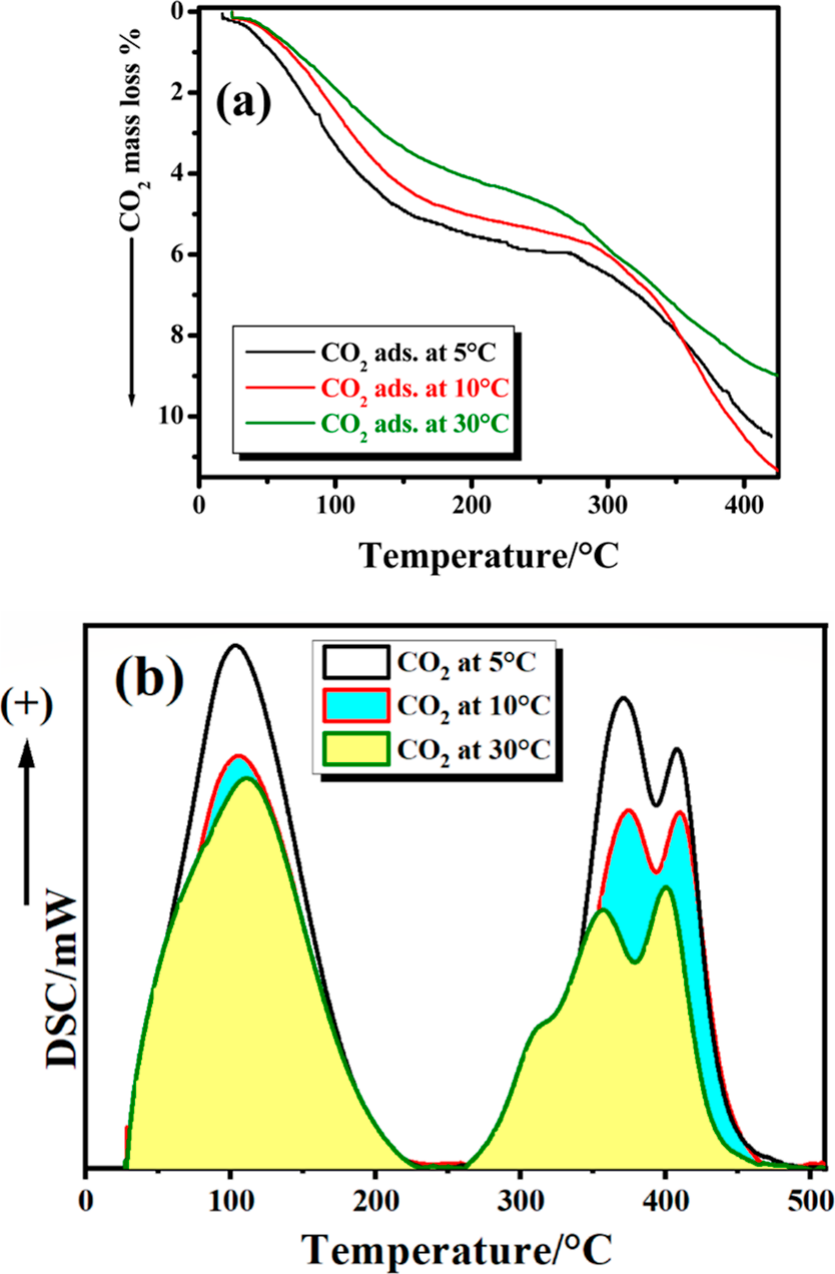
CO_2_ capture at different temperatures by Mg(OH)_2_-20% F, calcined at 400 °C, in a dynamic mode using a CO_2_ flow rate of 20 mL·min^–1^ (a,b).

**Figure 7:**
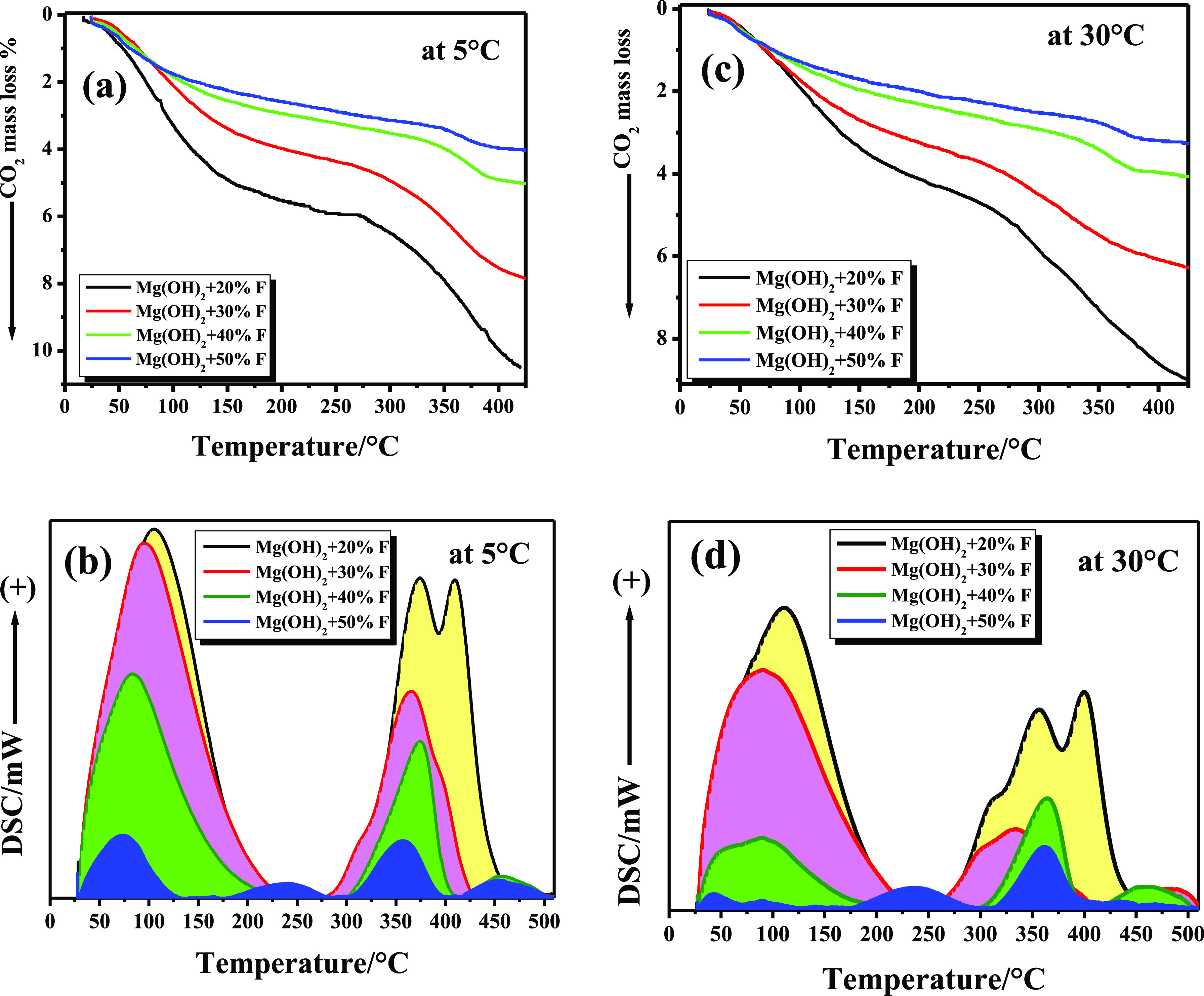
Capture of CO_2_ in a dynamic mode, using a flow rate of 20 mL·min^–1^, by Mg(OH)_2_ + *x*% F (wt/wt) calcined at 400 °C for 3 h in air, at 5 °C (a,b), and at 30 °C (c,d).

**Figure 8:**
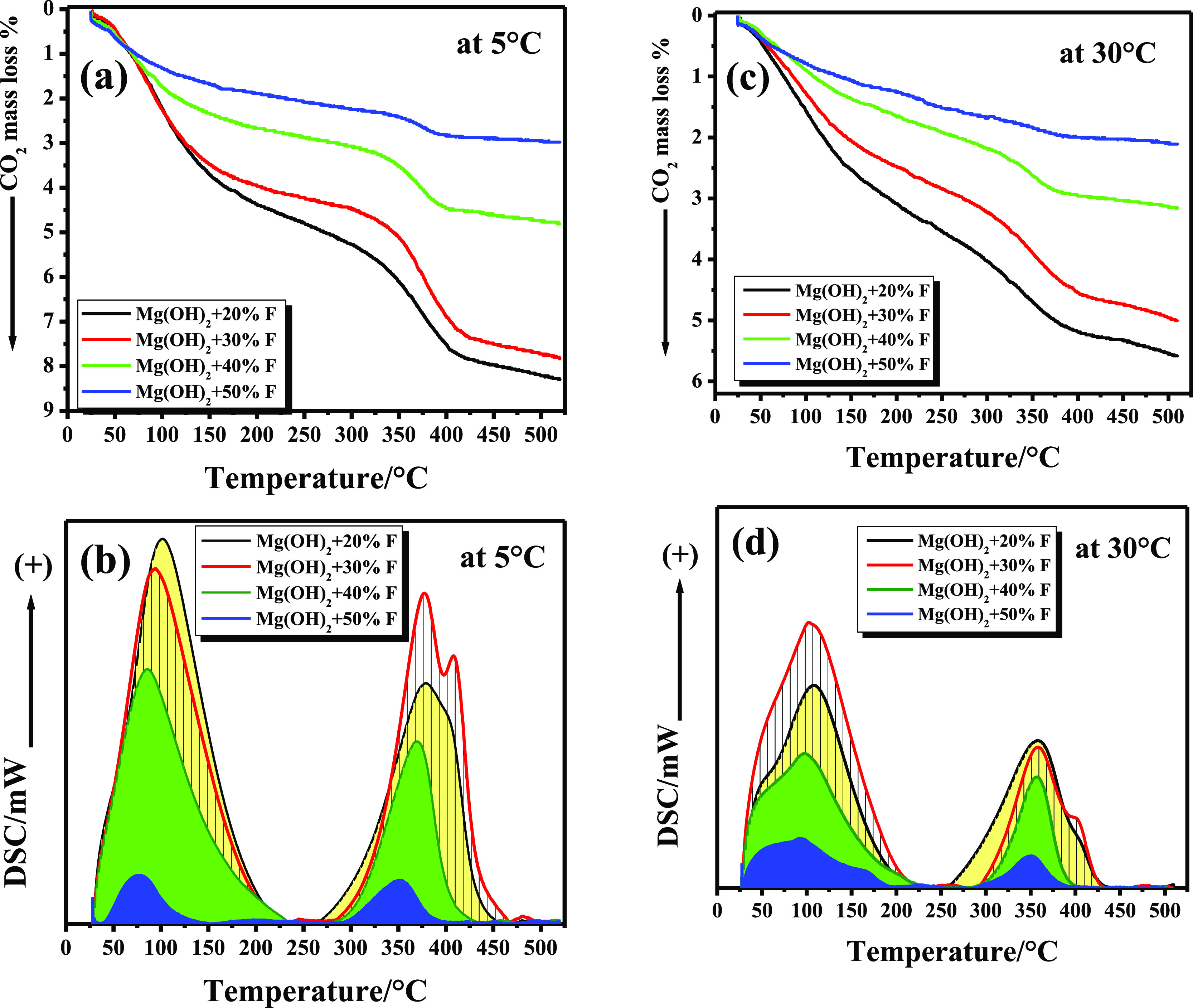
Capture of CO_2_ in a dynamic mode, using a flow rate of 20 mL·min^–1^, by Mg(OH)_2_ + *x*% F (wt/wt) calcined at 500 °C for 3 h in air, at 5 °C (a,b), and at 30 °C (c,d).

In order to adjust the temperature at which CO_2_ capture experiments yield logical and good results. A preliminary test was carried out at three different temperatures, that is, 5, 10, and 30 °C. TG- and DSC-TPD profiles (Figure 6) represent the results where CO_2_ permeation of the sample under test, that is, Mg(OH)_2_-20% F at 400 °C, with the highest surface area using a slow flow rate (20 mL·min^–1^) of CO_2_ in a vertical U-tube reactor is conducted. Two different temperatures, 5 and 30 °C, were selected for all CO_2_-capturing tests due to the overlap of results recorded at 10 °C with those at 5 °C, especially at temperatures <350 °C. At 5 °C, the sample captured 103.9 mg_CO_2__·g^–1^, which is equivalent to 2.36 mmol_CO_2__·g^–1^, while at 10 °C, it captured 111.9 mg_CO_2__·g^–1^ and 2.54 mmol_CO_2__·g^–1^, respectively. Furthermore, at 30 °C, the results were clearly different, as shown in Table 3. This test shed light on the efficiency of our samples in capturing CO_2_ at low and relatively high temperatures, that is, 30 °C, as will be discussed later. Figures 7 and 8 exhibit the collected TG and DSC-TPD profiles of CO_2_ captured by Mg(OH)_2_-*x* % F samples calcined at 400 and 500 °C at 5 and 30 °C, respectively. Table 3 lists the total amounts of CO_2_ captured by each sample as % mass loss (due to the desorption of CO_2_) in milligrams of CO_2_ per gram and millimoles of CO_2_ per gram, respectively, in the temperature ranges of RT–420 °C and RT–520 °C, in the case of samples calcined at 500 °C.

**Table 3:** CO_2_ Capture Capacity at 5 and 30 °C, Calculated Based on TG-TPD Curves, of Mg(OH) + *x* % F Adsorbents Calcined, for 3 h in Air, at 400 and 500 °C^a^

	calcined at 400 °C			calcined at 500 °C					
adsorbent	total % mass loss (RT–420 °C)	*total mg_CO_2__·g^–1^(RT–420 °C)	**total (mmol_CO_2__·g^–1^)(RT–420 °C)	total % mass loss (RT–420 °C)	*total mg_CO_2__·g^–1^(RT–420 °C)	**total (mmol_CO_2__·g^–1^)(RT–420 °C)	total % mass loss (RT–520 °C)	total mg_CO_2__·g^–1^(RT–520 °C)	total (mmol_CO_2__·g^–1^)(RT–520 °C)
CO_2_ Capture at 5 °C									
Mg(OH)_2_ + 20% F	10.39	103.9	2.36	7.73	77.3	1.76	8.21	82.1	1.86
Mg(OH)_2_ + 30% F	7.76	77.6	1.76	7.28	72.8	1.65	7.78	77.8	1.77
Mg(OH)_2_ + 40% F	4.77	49.5	1.13	4.47	44.7	1.02	4.73	47.3	1.07
Mg(OH)_2_ + 50% F	3.94	39.6	0.90	2.78	27.8	0.63	2.89	28.9	0.66
CO_2_ Capture at 30 °C									
Mg(OH)_2_ + 20% F	8.94	89.4	2.03	5.24	52.4	1.19	5.56	55.5	1.26
Mg(OH)_2_ + 30% F	6.20	61.9	1.41	4.64	46.4	1.05	4.99	49.9	1.13
Mg(OH)_2_ + 40% F	4.00	39.9	0.91	2.97	29.7	0.68	3.14	31.4	0.71
Mg(OH)_2_ + 50% F	3.22	32.2	0.73	1.99	19.9	0.45	2.09	20.9	0.47

a *,** for comparison at the same temperature range (RT–420 °C).

Due to the continuous desorption of CO_2_ from weak and strong basic sites, all TG-TPD curves exhibited two continuous mass loss steps. This behavior is evident whether CO_2_ was captured at 5 or 30 °C. The total % mass loss due to desorption of CO_2_, in the range of RT–420 °C, dramatically decreased as the (*x*) % F added to Mg(OH)_2_ steadily increased. This phenomenon is correlated with the decrease in the surface area of these samples due to the formation of MgF_2_ in these nanocomposite mixtures, as shown in Table 1, and consequently a reduction of some of the total number of basic sites over the sample’s surface (see Table 2). Comparing the amount of CO_2_ desorbed within the same temperature range reveals an additional effect of calcination temperature on the CO_2_-capturing efficiency of the samples. Heating samples up to 420 °C, the total amount of desorbed CO_2_ (as mg_CO_2__·g^–1^ and mmol_CO_2__·g^–1^) for samples calcined at 400 °C were always higher than those calculated for samples at 500 °C in the same temperature range; see Table 3. Calcinating samples at 500 °C liberates OH groups from the surface of these mixtures^62^ and eliminates surface defects of MgO. This clearly reduces both weak and strong basic sites over all samples. From the data of CO_2_ capture at 5 and 30 °C shown in Table 3 over samples calcined at 400 and 500 °C and the curves (a,c) in Figures 7 and 8, it is evident that the amount of CO_2_ captured by any sample is inversely proportional to the temperature at which the capturing process is carried out. This is ascribed to the exothermic nature of the adsorption of CO_2_ over the MgO surface, as confirmed recently.^23^

DSC-TPD profiles, as presented in Figures 7 and 8 (profiles b,d), revealed that our samples mainly include two different basic sites. Weak basic sites are observed in the temperature range of 29–200 °C, where CO_2_ is associated with these weak sites in the form of bicarbonate species.^54^ Furthermore, strong basic sites are recorded at a higher temperature range,^62,63^ that is, 290–450 °C, where CO_2_ interacts with the strong basic sites to form monodentate and bridged carbonate carbonates.^54,62^ This is clearly exhibited in the case of Mg(OH)_2_-20% F calcined at 400 °C and to somewhat in the case of the other samples, see Figure 7.

From the above-discussed results, one can conclude that the most active sample in capturing CO_2_ at 5 and 30 °C was Mg(OH)_2_-20% F, whether calcined at 400 or 500 °C. Its reactivity could be associated with the presence of surface interfaces between both MgF_2_ with low concentrations distributed in the bulk of MgO throughout its high surface area.

The calculated values of the molar enthalpy change (Δ*H*, J mol^–1^) corresponding to each desorption step, based on TG- and DSC-TPD profiles in Figures 7 and 8, for the most active adsorbent Mg(OH)_2_-20% F are listed in Table 4. It is obvious from Table 4 that values of Δ*H* calculated for step-1, in the case of Mg(OH)_2_-20% F at 400 °C, are always higher than the corresponding values calculated for step-2 during CO_2_ capture at 5 and 30 °C. This agrees with the desorbed amounts of CO_2_ in each step, see Table 4. This behavior does not match with that of the sample calcined at 500 °C. Our values of Δ*H* for both steps, as presented in Table 4, for desorption of CO_2_ after (1 h) capturing the pure gas, are in close agreement with those recently published.^12^

**Table 4:** Enthalpy Changes Corresponding to the Desorption Steps of CO_2_ Captured at 5 and 30 °C, Calculated Based on TG- and DSC-TPD Profiles, of the Most Active Adsorbent Mg(OH)_2_ + 20% F Calcined, for 3 h in Air, at 400 and 500 °C

	Mg(OH)_2_ + 20% F at 400 °C						Mg(OH)_2_ + 20% F at 500 °C					
	step-1(29–225 °C)			step-2(290–450 °C)			step-1(29–225 °C)			step-2(290–450 °C)		
experimental condition	Desor. CO_2_mg_CO_2__·g^–1^	Δ*H*_1_(J·mol^–1^)	*T*_max1_(°C)	Desor. CO_2_mg_CO_2__·g^–1^	Δ*H*_2_(J·mol^–1^)	*T*_max2_(°C)	Desor. CO_2_mg_CO_2__·g^–1^	Δ*H*_1_(J·mol^–1^)	*T*_max1_(°C)	Desor. CO_2_mg_CO_2__·g^–1^	Δ*H*_2_(J·mol^–1^)	*T*_max2_(°C)
CO_2_ capture at 5 °C	55.2	599.5	105	41.8	561.1	374, 410	49.9	528.5	101	27.0	572.6	379
CO_2_ capture at 30 °C	43.1	825.1	111	41.0	503.8	357, 400	32.6	526.3	108	15.3	584.1	359

Finally, Table 5 compares the CO_2_-capturing capacities of some adsorbents, under the same conditions, from previously published articles to the obtained results using the most active sample Mg(OH)_2_-20% F calcined at 400 and 500 °C.

**Table 5:** Comparison of CO_2_ Adsorption Capacity by Different MgO Adsorbents at Different Conditions as Cited in the References, and the Obtained Values of Our Adsorbent Mg(OH)_2_-20% F Calcined at 400 and 500 °C as the Most Active One^a^

		experimental conditions			
adsorbent	*S*_BET_m^2^·g^–1^	temp. (°C)	CO_2_(vol %)	mg_CO_2__·g^–1^ and mmol_CO_2__·g^–1^	reference
Mg(OH)_2_-20%F-400 °C	153.22	at 5	100%	103.9 mg_CO_2__	this work
				2.36 mmol_CO_2__	
		at 30	100%	89.4 mg_CO_2__	this work
				2.03 mmol_CO_2__	
	103.06	at 5	100%	82.1 mg_CO_2__	this work
Mg(OH)_2_-20%F-500 °C				1.86 mmol_CO_2__	
		at 30	100%	55.5 mg_CO_2__	this work
				1.26 mmol_CO_2__	
metal–organic framework (MOF)	N.C.	at 25	100%	114.96 mg_CO_2__	(64)
glass industry waste					
waste SiO_2_	16.82	at 20	50%	0.71 mmol_CO_2__	(65)
waste Al_2_O_3_	7.28	at 20	50%	0.68 mmol_CO_2__	(65)
*MgO	350.0	at 30	100%	30.0 mg_CO_2__	(66)
*MgO-APTES	134.0	at 30	100%	65.7 mg_CO_2__	(66)
*MgO-DETA	91.0	at 30	100%	47.7 mg_CO_2__	(66)
*MgO-PEI	72.0	at 30	100%	23.9 mg_CO_2__	(66)
**MgO-SR adsorbent	100.0	at 200	10%	2.39 mmol_CO_2__	(67)
CeO_2_	333.0	at 30	100%	10.4 wt %	(68)

a N.C.-not calculated, *MgO modified with 3-aminopropyl-triethoxysilane (APTES), diethylenetriamine (DETA), and polyethylenimine (PEI), **Prepared by a solid-state reaction.

The performance of our CO_2_ capture adsorbents appears to be superior to that of many adsorbents previously evaluated for the same application (see Table 5). This adsorbent includes MgO modified with 3-aminopropyl-triethoxysilane (APTES), diethylenetriamine (DETA), and polyethylenimine (PEI), MgO prepared by a solid–state reaction, and CeO_2_. This adsorbent also includes adsorbents derived from waste materials, such as “glass industry waste: waste SiO_2_ and waste Al_2_O_3_,” for the circular economy and waste management approach.^65−68^ Therefore, it is not surprising that our samples, which were prepared by mixing Mg(OH)_2_ with different wt % of NH_4_F·HF, particularly those containing 20–30% F, whether calcined at 400 or 500 °C, demonstrated a high capacity in capturing a large number of CO_2_ molecules in a short period of time (1 h), see Table 3, at different temperatures of 5 and 30 °C.

## Conclusions

1 Using environmentally friendly materials, such as Mg(OH)_2_ and NH_4_F·HF, a direct and simple method has been developed for the preparation of nanocomposite mixtures of Mg(OH)_2_-(*x*) wt % F.
2 The obtained samples were mixtures of MgO and MgF_2_ with a high surface area and a mesoporous structure. Using the DSC-TPD technique with pyrrole, all samples, whether calcined at 400 or 500 °C, exhibited two surface basic sites as weak and strong basic sites, as measured by pyrrole as a probe molecule.
3 We correlated the high efficiency of these samples toward CO_2_ capture at 5 and 30 °C to their high surface area, the high population of surface basic sites with weak and strong basic sites, and the presence of a low concentration of MgF_2_ composing active interfaces in MgO as the main component in these samples, as seen from XRD patterns. On the other hand, the efficiency was inversely proportional to the temperature at which the samples were calcined.
4 The Mg(OH)_2_-20% F sample was the most active in our investigation because it had a larger surface area and a greater total number of basic sites than the other samples (30–50% F). Consequently, the Mg(OH)_2_-20% F sample is the most effective in capturing CO_2_.

Future work will utilize the prepared Mg–O–F samples from this study and design MgO-based looping new systems for CO_2_ capture in the flue gas of diluted CO_2_ concentration and containing steam (H_2_O) and gas impurities (e.g., SO_2_, NO, CO, and HCl), which are commonly present in coal plants and other industrial applications, to investigate the effects of these impurities on the sorption performance. In addition, the future work will include increasing the operating pressure from 1 to 20 or 30 atm and the temperature to various values to examine the effect of increasing pressure and temperature on the sorption performance.
